# 

**Published:** 1914-05-02

**Authors:** 


### Appointments.

Mr. Cecil Rowntree, F.R.C.S., has been appointed surgeon to the Cancer Hospital, Fulham Road, S.W.

Mr. W. P. Hogg, M.B., Ch.B. (Aberd.), has been appointed house surgeon; Mr. George Wilson, M.B., Ch.B. (Edin.), assistant house surgeon, has been appointed second house surgeon; and Mr. I. J. Balkin, M.B., Ch.B. (Edin.), assistant house physician, all at Leicester Royal Infirmary.

The following appointments have been made at the Hampstead General and North-West London Hospital:—Mr. A. S. C. Courts, M.D. (Lond.), resident medical officer; Mr. H. Austin Williams, B.A. (Cantab.), M.R.C.S., L.R.C.P., house physician; and Mr. J. Z. Truter, M.B., Ch.B. (Edin.), house surgeon.

The following appointments have been made at Ancoats Hospital, Manchester: Mr. Charles Edgar Lea, M.D., Ch.B. (Vict.), M.R.C.P. Lond., honorary physician, and Mr. Harry Platt, M.S., M.B. Lond., F.R.C.S. Eng., honorary surgeon, to fill the vacancies caused through the appointment of Dr. C. H. Melland and Mr. W. H. Hey on the staff of the Manchester Royal Infirmary.

### Estate of the First Woman to Inhale Chloroform.

Mrs. Agnes Waugh Thomson, of Mount Ephraim Road, S.W., said to have been the first woman to inhale chloroform, who died on January 4, aged eighty-three years, daughter of Commander Petrie, has left estate of which £3,130 is net personalty.

### Well-known Hospital Treasurer's Estate.

Sir James John Trevor Lawrence, second baronet, K.C.V.O., of Prince's Gate, S.W., and of Burford Lodge, Dorking, Surrey, formerly of the Indian Medical Service and M.P., for twelve years treasurer of St. Bartholomew's Hospital, has left unsettled property, of which the net personalty amounts to £11,732. He left to his wife the use for life of his art collection, including his Japanese lacquers and enamels and his Oriental, English, and other European porcelain, with remainder to his residuary estate, and requested that she would give to the Royal Gardens at Kew any of his collection of plants as should be mainly of botanical interest. He was for twenty-eight years president of the Royal Horticultural Society.

### A Racehorse Owner and the Hospitals.

Mr. Ernest Dresden, of London and of Newmarket, a racehorse owner, who directed that his remains should be cremated, has left £5,0C0 to the Middlesex Hospital, and £1,000 each to the Rous Memorial Hospital, Newmarket; the County Hospital, Bury St. Edmunds; the Infant Orphan Asylum, Wanstead; the Cancer Hospital, Fulham Road, S.W.; and the Hospital for Children, Great Ormond Street, W.C.; and large bequests to servants.

A lead to the effacement of the outstanding debt on the building of the reconstructed Children's Hospital at Leicester has been given by the High Sheriff of Leicestershire, who has contributed £100 to the fund.

### Our Prize Scheme.

**Owing to the number of replies, with drawings, received in the Ward Locker Competition, and the consequent unavoidable delay in selecting the best six designs and making the blocks, we regret we are unable to publish the illustrated descriptions in this issue, but hope to do so next week, when the necessary voting paper and full particulars of the method of recording votes will also be published.**

### Gifts and Bequests.

THE QUEEN has sent a gift of wild flowers to the Chelsea Hospital for Women.

AMONG the latest contributions received for King Edward's Hospital Fund for London is an annual subscription of £500 from Messrs. N. M. Rothschild and Sons.

MRS. MARY ANN MACKINLAY, of Hove, Sussex, who died on March 8, in her 100th year, has left, subject to her daughter-in-law's interest, £100 to the Royal Free Hospital.

MR. FREDERICK BURTON, of Pendleton, Manchester, has left £2,000 to the Salford Royal Hospital, and £1,000 each to the Manchester Royal Infirmary and St. Mary's Hospital, Manchester, and £500 to the Denbigh Infirmary.

MRS. MARGARET CHEVERS, of Portrunna, Co. Galway, has left £1,000 to the Victoria Eye and Ear Hospital, Dublin. After other bequests she left the residue of her estate in equal shares to the Jervis Street Hospital, the Mater Misericordiæ Hospital, and St. Vincent's Hospital, all of Dublin.

THE Committee of the Cambridge Research Hospital are making a special effort to raise an endowment fund of £10,000, and the executors of the late Sir William Dunn nave sent a donation of £2,500.

THE Leicester Royal Infirmary has received legacies from the undermentioned estates: £200 from Mr. John Norman, £150 from Mrs. Jane Barker, £100 from Mr. Thomas Berridge, £50 from Miss Annie Harding, £20 from Miss Mary Boyer, and £20 from Mrs. Elizabeth Crockerill.

MR. HENRY SKEATE WHITE, of Stockwell Park Road, S.W., and of Kennington Road, S.E., estate agent, has left £200 each to the Royal Ear Hospital, Dean Street, Soho, the Royal Dental Hospital, Leicester Square, the Royal Hospital for Incurables, Putney, and to the British Home and Hospital for Incurables, Streatham.

MR. THOMAS SHERREN WHITTAKER, of Broadstairs, and the Temple, barrister-at-law, also in business as a tailor, has left £250 to the Kent Rugby Union, and the ultimate residue as to one-half to Guy's Hospital in memory of his parents, one-half to found and endow a cottage hospital for poor persons resident in Broadstairs or the Isle of Thanet.

---

MR. TREVOR JOHN PRITCHARD, M.B., C.M., L.R.C.P., of Bognor, Sussex, retired surgeon, has left estate valued at £9,811.

DR. BENJAMIN NEALE DALTON, M.D., M.R.C.S., L.R.C.P., of Adelaide Road, Hampstead, N.W., formerly of South Norwood, has left estate valued at £20,110.

### The Book Market.

LIST OF NEW BOOKS RECEIVED FROM THE FOLLOWING PUBLISHERS:—

J. Wright and Sons: "Appendicitis." By Edmund Owen, F.R.C.S. 3s. 6d. net.

Vigot, Paris: "Tuberculose Pulmonaire." By Dr. J. F. Larrieu. Price 2 francs.

Mills and Boon: "An Absent Hero." By Mrs. Fred Reynolds. 6s. net.

T. R. Blumer, Sunderland: "Hospital Prayers."

### Institution and Public Reports.

A Short Account of the Orphans' Home, Austral Street, West Square, London, S.E., being the Forty-seventh Annual Report by Charlotte Sharman. London: Hudson and Kearns, Ltd., Hatfield Street, S.E. Price 2d.

Annual Report for 1913 on the Health of Weston-super-Mare, by John Wallace, M.B., C.M., B.Sc., M.O.H., and Medical Superintendent of the Isolation Hospital: to which is appended the Report of the Sanitary Inspector and the Meteorological Report.

Fourth Report for 1912 and 1913 of St. George's Dispensary and School Clinic, Surrey Row, Blackfriars Road, S.E.

Report (1913) of the Edinburgh Dental Hospital and School, 31 Chambers Street.

Sixty-ninth Annual Report of the Birmingham and Midland Ear and Throat Hospital.

The Thirty-second Annual Report (for 1913) of the Hampstead General and North-West London Hospital.

The Annual Report (for 1913) of the South London Hospital for Women, Clapham Common, S.W. Out-patient Department, 88 and 90 Newington Causeway, S.E.

The Seventieth Annual Report (for 1913) of Ayr County Hospital.

### Pamphlets and Papers.

Observations on the Working of Sanatorium Benefit, by S. Vere Pearson, M.D., M.R.C.P.

Rates of Subscription: Twelve months, 6/6; Six months, 4/-; Three months, 2/-, post free to any address in the United Kingdom. Abroad, Twelve months, 8/8; Six months, 5/-; Three months, 2/6, post free.—Address The Subscription Dept., "The Hospital," The Hospital Building, 28/29 Southampton Street, Strand, London, W.C.

